# Therapeutic Reduction Mammoplasty: Experience of a Single Institute

**DOI:** 10.7759/cureus.33603

**Published:** 2023-01-10

**Authors:** Marta Azevedo, Carolina Chaves, Gustavo Coelho, Carolina Andresen, Augusta Cardoso, Horacio Costa

**Affiliations:** 1 Plastic and Reconstructive Surgery, Centro Hospitalar Vila Nova de Gaia/Espinho, Vila Nova de Gaia, PRT

**Keywords:** symmetrization mammaplasty, reconstructive breast surgery, therapeutic mammoplasty, oncoplastic breast surgery, breast cancer management

## Abstract

Introduction: Breast cancer is the most common malignancy in women worldwide as reported by the World Health Organization. The concept of oncoplastic breast surgery appeared as an extension of breast-conserving surgery, applying breast reduction techniques with more acceptable aesthetic and functional outcomes. The purpose of the present study was to describe the breast cancer population of a single institute submitted to lumpectomy and bilateral immediate breast reduction or mastopexy and its complications.

Material and Methods: This is a retrospective observational study including patients submitted to lumpectomy and immediate bilateral breast reduction or mastopexy. Patients and tumour characteristics, surgical technique, complications, follow-up period, and recurrence data were obtained and analyzed.

Results: A total of 49 patients were submitted to lumpectomy and bilateral breast therapeutic reduction/mastopexy, with a mean age of 56.47 ±8.58 years and a mean body mass index of 28.68kg/m^2^ ±3.94 kg/m² between January 2019 and December 2021. Invasive tumours of no specific type, associated or not, with carcinoma intraductal in situ were the most common histological type corresponding to almost 80% of the cases with T1 stage corresponding to more than half of the cases. Sixteen percent of the patients had early minor complications with wound dehiscence associated with wound delayed healing, corresponding to 75% of the cases. Body mass index had a statistical difference between groups (p=0,006, t-test).

Conclusions: The low rates of minor and major complications show that immediate therapeutic breast reduction can be a suitable approach in selected cases.

## Introduction

As reported by the World Health Organization, breast cancer is the most common malignancy in women worldwide contributing to over 25% of new cancer cases diagnosed in 2020 (excluding non-melanoma skin cancer) [1]. In Portugal, 7041 new cases were diagnosed causing 1864 deaths in 2020 [2].

The concept of oncoplastic breast surgery (OBS) appeared as an extension of breast-conserving surgery (BCS) [3]. Strategies of volume displacement (breast reduction and mastopexy) and volume replacement (implant-based reconstruction/flap reconstruction) were introduced in order to improve the final result [4]. By applying breast reduction techniques to the field of breast cancer surgery, therapeutic breast mammoplasty can achieve more acceptable aesthetic and functional outcomes by reducing breast size, minimizing the significant impact of radiotherapy and achieving a preferred breast size and shape [5]. It also allows wide margins of excision improving oncological safety [6] and is associated with fewer aesthetic complications [7].

Although surgical complications after aesthetic breast surgery procedures can be associated with unfavourable outcomes, complications in breast reconstruction procedures can cause morbidity in several ways, the most important being the delay of subsequent oncological treatments [8]. Risk factors that seem to be associated with local complications after breast reduction procedures include obesity [9], resection weight [10], smoking [11], and increased age [12]. In oncoplastic surgery, the possible role of neoadjuvant chemotherapy in postoperative complications is still unknown [13].

The purpose of this study is to describe the breast cancer population of a single institute submitted to lumpectomy and bilateral immediate breast reduction or mastopexy, as well as its associated complications.

## Materials and methods

This is a retrospective observational study that analyzed patients submitted to lumpectomy and immediate bilateral breast reduction or mastopexy between January 2019 and December 2021 (36 months) at Centro Hospitalar Vila Nova de Gaia/Espinho (CHVNG/E), Vila Nova de Gaia, Portugal. Patients submitted to other types of oncoplastic procedures, unilateral procedures, or procedures associated with benign lesions were excluded from this study. Patients’ information and medical records were obtained through the hospital electronic system and database. 

The data collected included age at presentation, comorbidities, body mass index (BMI), smoking history, tumour location, size, and stage, surgical technique, breast weight excision and margins, neoadjuvant and adjuvant treatments, outcomes, and recurrence. Tumour size and stage were based on pathological specimens. For the patients undergoing neoadjuvant therapy, tumour size was classified on the basis of initial pretreatment mammographic tumour size.

Outcomes assessed included immediate complications, early complications (first three months postoperatively after discharge) and secondary surgeries. Immediate complications were categorized as major if they had caused a reintervention or additional treatment prolonging the hospitalization time. These patients were operated on by a surgical team composed of oncological and plastic surgeons; oncological surgeons performed all lumpectomies and sentinel node biopsy/lymph node dissection and plastic surgeons performed the breast therapeutic and symmetrization mammoplasty.

Descriptive analysis of patient characteristics was performed using IBM SPSS Statistics for Windows, Version 26.0 (Relesed 209; IBM Corp., Armonk, New York, United States). Continuous variables were assessed for normality (Shapiro-Wilk test or skewness and kurtosis) and described using mean and standard deviation or median and interquartile range, accordingly. Categorical variables were described using absolute and relative frequencies. The group with postoperative complications and the group without complications were compared. Normality was assessed in each group. Normally distributed continuous variables were compared between the two groups using Student’s t-test for independent samples. Categorical variables were compared using Pearson’s Chi-squared test or Fisher’s exact test, depending on expected counts. A significance cut-off value of 0.05 was used.

## Results

During the three-year period, a total of 49 patients underwent lumpectomy/quadrantectomy and bilateral breast therapeutic reduction/mastopexy. Patient-related and oncological variables are described in Table 1.

**Table 1:** Patients characteristics and comorbidities and tumour location, stage, and histological subtype DCIS: ductal carcinoma in situ; LCIS: lobular carcinoma in situ *The mean patient age at the time of surgery

Number of patients	49
Age (SD) years *	56.47 (8.58)
Comorbidities	
Arterial hipertension	19 (38.8%)
Diabetes mellitus	9 (18.4%)
Dislipidemia	17 (34.7%)
Immunosuppression therapy	1 (2%)
Smoking status	
Smoker/ex-smoker	4 (8.2%) /5 (10.2%)
Non-smoker	40 (81.6%)
BMI (kg/m2)	28.68 (3.93)
Size of invasive tumour, mean (SD), mm (n=45)	18.06 (11.58)
Laterality	
Right	18 (36.7%)
Left	30 (61.2%)
Bilateral	1 (2%)
Histological subtype (n=50)	
DCIS	5 (10%)
Invasive breast carcinoma of no special type	23 (46%)
Invasive breast carcinoma of no special type+DCIS	17 (34%)
Invasive lobular carcinoma	3 (60%)
Invasive lobular carcinoma + LCIS	1 (2%)
Invasive lobular carcinoma+Invasive of no special type	1 (2%)
c/p stage (t) (n=50)	
Tis	5 (10%)
T1a	3 (6%)
T1b	11 (22%)
T1c	15 (30%)
T2	14 (28%)
T3	2 (4%)

The mean patient age at the time of surgery was 56.47 ±8.58 years. The comorbidities included arterial hypertension (n=9, 38.8%), dyslipidemia (n=17, 34.7%), thyroid pathology (n=10, 20.4%), type 2 diabetes mellitus (n=9, 18.4%), and one case of liver transplantation history with immunosuppression therapy at the time of surgery. Nine patients (18.4%) had a history of prior or ongoing smoking. The mean BMI was 28.68kg/m2 ± 3.94 kg/m². Although all patients had bilateral procedures, 61% (n=30) had left-sided breast lesions, 36.7% (n=18) had right-sided breast lesions, and one case was bilateral.

Invasive tumours of no special type associated or not with carcinoma intraductal in situ were the most common histological type corresponding to 80% of the cases (n=40). The mean size of invasive tumours was 18.06 mm (11.58), present in 44 patients; T1 (size:0.5-2cm) tumours corresponded to 58% of the cases, in which five patients did neoadjuvant therapy previously to surgery; 86% of the T2 (2-5cm) patients (n=14) also did neoadjuvant therapy before the indication for breast reduction mammoplasty.

All patients were submitted to oncoplastic surgery with lumpectomy/quadrantectomy and therapeutic breast reduction/mastopexy. Tumour and additional ipsilateral breast mean weight was 295 g (range 35-685g). Contralateral breast reduction or mastopexy ensued, with excision of a similar amount of breast tissue in order to obtain symmetry (mean 310.8 g, range 15-820 g). Regarding tumour location, 57% (n=28) of the lesions were located in the upper breast quadrants (upper inner quadrant, transition of the upper quadrants, upper outer quadrant), which is in line with the fact that in 65% of the patients, a bilateral breast mammoplasty was performed with an inferior/modification of the inferior pedicle. Associated with the surgery, in 14.3% (n=7) of the patients did axillary clearance and the rest (n=43) were submitted to sentinel node biopsy.

Neoadjuvant chemotherapy was performed in 28.6% (n=14) and adjuvant chemotherapy in 32.6% (n=16), four of these patients had both. 91.8% (n=45) of the patients were assigned to postoperative radiotherapy. The patients that needed radiotherapy without adjuvant chemotherapy had a mean started time of 74.4 days after surgery. When adjuvant chemotherapy was decided, the radiotherapy started with a mean of 159 days after surgery. Hormonotherapy was used in 81.6% (n=40) when hormonal receptors were positive. Information regarding tumour location, surgical procedure, and other oncological treatments performed are summarized in Table 2.

**Table 2:** Breast tumour location, surgical characteristics, and other oncological treatments

Quadrantectomy/ Lumpectomy location (n=50)	
Lower outer quadrant	3 (6%)
Lower inner quadrant	2 (4%)
Upper outer quadrant	18 (36%)
Upper inner quadrant	6 (12%)
Outer quadrant transition	5 (10%)
Inner quadrant transition	2 (4%)
Upper quadrant transition	5 (10%)
Lower quadrant transition	7 (14%)
Multifocal - more than one location	1 (2%)
Retroareolar	1 (2%)
Pedicle	
Inferior	27 (55.1%)
Inferolateral	4 (8.2%)
Superomedial	10 (20.4%)
Superior	3 (6.1%)
Vertical bipedicle	1 (2%)
Posteroinferior	1 (2%)
Different pedicles between breasts	3 (6.1%)
Mean (bilateral) weight of the ressected breasts (SD)	295 (173.6)
Other oncological treatments	
Sentinel node biopsy	44 (89,9%)
Axillary clearance	7 (14,3%)
Neoadjuvant chemotherapy	10 (20,4%)
Adjuvant chemotherapy	12 (24,5%)
Both (neoadjuvant+adjuvant chemotherapy)	4 (8,2%)
Hormonotherapy	40 (81,6%)
Radiotherapy	46 (93,9%)

In terms of complications, 16% of the patients had early minor complications and wound dehiscence associated with delayed healing of wound was the most common (75% of the cases). Three patients (37.5%) needed surgical intervention with partial thickness skin graft to close the wound (Table 3).

**Table 3:** Postoperative complications

Complications	
Infection	1 (2%)
Haematoma with wound dehiscence	1 (2%)
Wound dehiscence/delayed wound healing	6 (12%)

When comparing patients with and without complications, smoking, age, quantity of tissue resected, and BMI were analyzed. Smoking, age, and quantity of tissue resected seem to have no association with the presence of complications (smoking: p=1, Fisher Exact Test; age: p=0.68, t-test; quantity of tissue resected: p=0.095, t-test). On the contrary, BMI had a statistical difference between groups with wound dehiscence/delayed wound healing happening only in patients with BMI higher than 32 kg/m^2^ (p=0.006, t-test) (Table 4).

**Table 4:** Possible risk factors for postoperative complications

	No complications	Complications	p-value
Age (mean)	56,24	57,63	0,68
Smoking (yes/no)	8/33	1/7	1
Weight of excised breast (mean)	280	392	0,095
BMI (mean)	28	32,1	0,006

During the follow-up period, one patient died due to a mental health problem and another patient had a recurrence of the disease 16 months after breast oncoplastic surgery and axillary clearance.

## Discussion

Oncoplastic breast surgery has been defined as an oncologic-aesthetic-functional individualized surgical approach as it can improve the general indications for breast-conserving surgery without compromising the aesthetics or the oncological outcomes and improving quality of life [2,14]. Besides providing an aesthetic and oncological better outcome, it also makes the radiation dose homogeneity possible in larger breasts, as well as more tolerance and less radiation local side effects [15].

Although patients undergoing immediate oncoplastic breast reduction are usually older and with a higher number of comorbidities compared to standard reduction mammoplasty, they show a similar safety profile without significant delays to adjuvant therapy [16]. The same is not verified when comparing immediate and delayed breast therapeutic reduction complications when radiotherapy is performed. In this case, the rate of complications is known to be higher when the procedure is delayed [17].

Risk factors for complications after breast reduction surgery include smoking, obesity, breast resection volume, and advanced age [10]. Smoking has been associated with delayed wound healing due to a decrease in wound perfusion in many surgical procedures including breast surgery [18,19], although it seems to have no association with increased reoperation rates [10]. In this study, 8.2% of patients were active smokers when surgery was proposed; every patient was advised to stop smoking so that the reconstructive surgery could be performed safely. Specimen weight and complications had no relation in our study, although several studies in breast reduction surgery showed an association between both [10]. Age also seems to have an association with complications in breast reduction. Mixed result are seen, with studies revealing an association with advanced age (more than 50 years old) due to the decrease of estrogen levels [12], or an inverse association, with younger age being more common [10]. In this study, patients’ mean age is 56.47 (±8.58) years old, which makes it difficult to have a conclusion related to this point. The implications of BMI in the context of breast reduction remain unclear. Some studies have demonstrated an increased risk of surgical site complications including delayed healing, seroma, infection, hematoma, and hypertrophic scarring [20]. In our study, all patients with wound dehiscence/delayed wound healing were obese and with a BMI greater than 32 kg/m^2^. 

The tumour location and the need for skin excision en bloc with the tumour dictate the design of the excision reduction pattern. For example, if the tumour is located outside the regular pattern, the concomitant skin excision would distort or alter the nipple-areolar complex shape or position. In this case, there is the need for technical modifications that will be subject to each centre’s experience [21]. When necessary, these modifications have to be done [22].

This study highlights that therapeutic breast reduction/mastopexy with symmetrization is a valuable surgical option for early breast cancer in patients with large and ptotic breasts to avoid poor outcomes from breast-conserving surgery and, in some cases, to avoid mastectomy. In our centre, it corresponded to 35% of all oncoplastic surgeries performed by the surgical team between 2019-2021.

This study has some limitations. These include its retrospective design with information collected by reports and resulting bias, and the inclusion of a limited number of patients with a small percentage of complications, which can give possible associations but can't allow the authors to confirm risk factors.

## Conclusions

The low rates of minor and major complications in this study show that breast therapeutic reduction with symmetrization, as a branch of breast-conserving surgery, is a suitable approach in selected cases. Some factors seem to have an association with complications in cosmetic breast reduction surgery, although less studied in oncoplastic surgery. In this study, increased BMI seems to have an association with wound healing complications, which should be taken into account when selecting patients, especially when complications can lead to the delay of adjuvant treatments.

## References

[1] Sung H, Ferlay J, Siegel RL, Laversanne M, Soerjomataram I, Jemal A, Bray F (2021). Global cancer statistics 2020: GLOBOCAN estimates of incidence and mortality worldwide for 36 cancers in 185 countries. CA Cancer J Clin.

[2] Bertozzi N, Pesce M, Santi P, Raposio E (2017). Oncoplastic breast surgery: comprehensive review. Eur Rev Med Pharmacol Sci.

[3] (2021). Portugal Fact Sheet: Globocan 2020.

[4] Chatterjee A, Gass J, Patel K (2019). A consensus definition and classification system of oncoplastic surgery developed by the American Society of Breast Surgeons. Ann Surg Oncol.

[5] Macmillan RD, James R, Gale KL, McCulley SJ (2014). Therapeutic mammaplasty. J Surg Oncol.

[6] Mohsen SM (2018). Therapeutic reduction mammoplasty techniques in management of breast cancer in large-breasted females - a comparative study between inferior and superior pedicle reduction mammoplasty. Egypt J Surg.

[7] Clough KB, Lewis JS, Couturaud B, Fitoussi A, Nos C, Falcou MC (2003). Oncoplastic techniques allow extensive resections for breast-conserving therapy of breast carcinomas. Ann Surg.

[8] Lin KY, Johns FR, Gibson J, Long M, Drake DB, Moore MM (2001). An outcome study of breast reconstruction: presurgical identification of risk factors for complications. Ann Surg Oncol.

[9] Simpson AM, Donato DP, Kwok AC, Agarwal JP (2019). Predictors of complications following breast reduction surgery: a National Surgical Quality Improvement Program study of 16,812 cases. J Plast Reconstr Aesthet Surg.

[10] Cunningham BL, Gear AJ, Kerrigan CL, Collins ED (2005). Analysis of breast reduction complications derived from the BRAVO study. Plast Reconstr Surg.

[11] Hillam JS, Borsting EA, Chim JH, Thaller SR (2017). Smoking as a risk factor for breast reduction: an analysis of 13,503 cases. J Plast Reconstr Aesthet Surg.

[12] Shermak MA, Chang D, Buretta K, Mithani S, Mallalieu J, Manahan M (2011). Increasing age impairs outcomes in breast reduction surgery. Plast Reconstr Surg.

[13] Lorentzen T, Heidemann LN, Möller S, Bille C (2022). Impact of neoadjuvant chemotherapy on surgical complications in breast cancer: A systematic review and meta-analysis. Eur J Surg Oncol.

[14] Urban C, Lima R, Schunemann E, Spautz C, Rabinovich I, Anselmi K (2011). Oncoplastic principles in breast conserving surgery. Breast.

[15] Moody AM, Mayles WP, Bliss JM (1994). The influence of breast size on late radiation effects and association with radiotherapy dose inhomogeneity. Radiother Oncol.

[16] Pawlak N, Karamchandani M, Wareham C (2022). Comparing oncoplastic breast reduction with immediate symmetry surgery to standard breast reduction surgery: are postoperative complications worse?. J Surg Oncol.

[17] Clough KB, Thomas SS, Fitoussi AD, Couturaud B, Reyal F, Falcou MC (2004). Reconstruction after conservative treatment for breast cancer: cosmetic sequelae classification revisited. Plast Reconstr Surg.

[18] Bartsch RH, Weiss G, Kästenbauer T, Patocka K, Deutinger M, Krapohl BD, Benditte-Klepetko HC (2007). Crucial aspects of smoking in wound healing after breast reduction surgery. J Plast Reconstr Aesthet Surg.

[19] Lewin R, Göransson M, Elander A, Thorarinsson A, Lundberg J, Lidén M (2014). Risk factors for complications after breast reduction surgery. J Plast Surg Hand Surg.

[20] Myung Y, Heo CY (2017). Relationship between obesity and surgical complications after reduction mammaplasty: a systematic literature review and meta-analysis. Aesthet Surg J.

[21] Andresen C, Cardoso A, Cunha C (2019). Therapeutic breast reduction—are doctors and patients satisfied?. Eur J Plast Surg.

[22] Cardoso A, Coelho G, Esteves J, Costa H (2021). Therapeutic breast reduction in upper quadrant breast tumor. Surg Case Rep.

